# Pyridoxal 5′-Phosphate-Dependent Enzymes at the Crossroads of Host–Microbe Tryptophan Metabolism

**DOI:** 10.3390/ijms21165823

**Published:** 2020-08-13

**Authors:** Barbara Cellini, Teresa Zelante, Mirco Dindo, Marina M. Bellet, Giorgia Renga, Luigina Romani, Claudio Costantini

**Affiliations:** 1 Department of Experimental Medicine, University of Perugia, 06132 Perugia, Italy; barbara.cellini@unipg.it (B.C.); teresa.zelante@unipg.it (T.Z.); marinamaria.bellet@unipg.it (M.M.B.); rengagiorgia@gmail.com (G.R.); luigina.romani@unipg.it (L.R.); 2 Okinawa Institute of Science and Technology Graduate University, 1919-1 Tancha, Onna-son, Okinawa 904-0412, Japan; MIRCO.DINDO@oist.jp

**Keywords:** tryptophan, microbiome, pyridoxal 5′-phosphate

## Abstract

The chemical processes taking place in humans intersects the myriad of metabolic pathways occurring in commensal microorganisms that colonize the body to generate a complex biochemical network that regulates multiple aspects of human life. The role of tryptophan (Trp) metabolism at the intersection between the host and microbes is increasingly being recognized, and multiple pathways of Trp utilization in either direction have been identified with the production of a wide range of bioactive products. It comes that a dysregulation of Trp metabolism in either the host or the microbes may unbalance the production of metabolites with potential pathological consequences. The ability to redirect the Trp flux to restore a homeostatic production of Trp metabolites may represent a valid therapeutic strategy for a variety of pathological conditions, but identifying metabolic checkpoints that could be exploited to manipulate the Trp metabolic network is still an unmet need. In this review, we put forward the hypothesis that pyridoxal 5′-phosphate (PLP)-dependent enzymes, which regulate multiple pathways of Trp metabolism in both the host and in microbes, might represent critical nodes and that modulating the levels of vitamin B6, from which PLP is derived, might represent a metabolic checkpoint to re-orienteer Trp flux for therapeutic purposes.

## 1. Introduction

**Figure 1:**
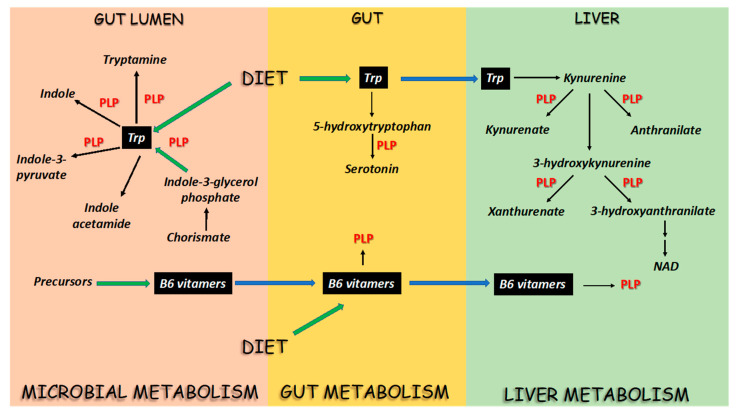
Schematic overview of the microbial and host tryptophan (Trp) and B6 vitamers metabolic pathways. The panel depicts the pathways of Trp and B6 vitamers metabolism in the gut microbiota (pink, left side) and in the host, with particular reference to the gut (yellow, central) and the liver (green, right side) metabolic pathways. The green arrows indicate the source or synthesis of Trp and B6 vitamers, the blue arrows indicate the transport among the different compartments, and the black arrows indicate the metabolic reactions. The panel highlights the distribution of pyridoxal 5′-phosphate (PLP)-dependent enzymes in the microbes and the host governing the synthesis of key Trp metabolites, emphasizing their dependence on B6 vitamer availability. NAD: Nicotinamide adenine dinucleotide. Details are described in the main text.

Tryptophan (Trp) is an aromatic amino acid characterized by the presence of a side chain indole, a bicyclic structure consisting of a benzene ring fused to a pyrrole ring [1]. The indole group is responsible for the peculiar structural and functional properties of Trp. Indeed, indole makes Trp a non-polar amino acid, which is important for stabilizing the structure of proteins, and is the scaffold of a series of Trp-derived metabolites with wide-ranging physiological activities [1] (Figure 1). Trp metabolites can be generated not only by the host, but also by other organisms that interact with the host. For instance, the microbiota, i.e., the commensal microbes that inhabit the surfaces exposed to the external environment, such as the skin and the respiratory, gastrointestinal, and genitourinary tracts, can generate bioactive Trp metabolites [2]. Two characteristics make Trp an intriguing molecule in the relationship between the host and other organisms. First, Trp is an essential amino acid in mammals and needs to be acquired from the diet, whereas, in general, bacteria, fungi, and higher plants express enzymes for Trp biosynthesis. Second, the Trp metabolic pathways and the resulting bioactive molecules can either be shared or differ between mammals and other organisms [3]. All these characteristics create a network of inter-dependency and connectivity, centered around Trp and molded by a plethora of Trp-derived metabolites, which is critical for both the regulation of host physiology and the modulation of the interaction between the host and other organisms. Indeed, a dysregulation of Trp metabolism has been associated with the occurrence of diverse pathological conditions and the therapeutic efficacy of balancing among the distinct fates of Trp metabolism is increasingly being recognized [4]. This therapeutic effort would benefit from a comprehensive assessment of the complexity of Trp metabolism in the host and microbes. For instance, what orientates the flux of Trp along the different metabolic pathways in physiological conditions? Is there a common denominator among all, or at least some, of the metabolic pathways? Is it possible to identify a checkpoint that coordinates the traffic of Trp? A biochemical investigation of Trp metabolism may provide an interpretative key to dissect this complexity.

**Figure 2:**
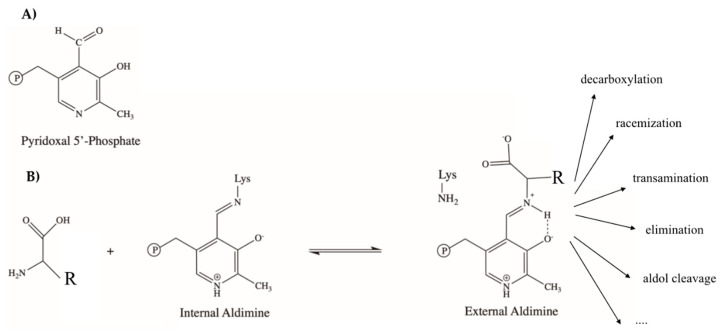
Schematic view of pyridoxal 5′-phosphate (PLP) and catalytic mechanism of PLP enzymes. (**A**) Chemical structure of PLP. (**B**) Schematic depiction of the reaction catalyzed by PLP-dependent enzymes with the possible outcomes.

Vitamin B6 includes a group of chemically similar compounds, or vitamers, that are derivatives of 2-methyl-3-hydroxy-5-hydroxymethyl-pyridine [5]. In the form of pyridoxal 5′-phosphate (PLP) (Figure 2A), vitamin B6 works as a co-factor for more than 140 enzymatic reactions, corresponding to ≈4% of all classified activities [6]. Interestingly, critical steps in the synthesis and metabolism of Trp in mammals and microbes are regulated by PLP-dependent enzymes. It is therefore likely that vitamin B6, from which the active form PLP is derived, might intersect the Trp metabolism to regulate its flux along the different metabolic pathways, thus working as a metabolic checkpoint.

In this review, we provide an overview of the metabolic dependence between vitamin B6 and Trp in mammals and microbes, then discuss how the regulation of Trp flux may occur in function of vitamin B6 levels, and finally propose how the modulation of vitamin B6 levels may have potential therapeutic implications in pathological conditions.

## 2. PLP-Dependent Enzymes in the Flux of Trp in Mammals and Microbes

As previously stated, Trp is essential in mammals while, in general, bacteria, fungi, and higher plants can synthesize Trp via the shikimate pathway, a seven-step metabolic route that collectively converts phosphoenol pyruvate and erythrose-4-phosphate into chorismate, a substrate for the three aromatic amino acids Trp, phenylalanine (Phe), and tyrosine (Tyr) [7]. In the Trp biosynthetic pathway, the subsequent action of four enzymes converts chorismate into indole-3-glycerol phosphate, which becomes the substrate of tryptophan synthase [8]. Tryptophan synthase is an α_2_β_2_ tetrameric PLP-dependent enzyme that catalyzes Trp synthesis, thus making the final step of the biosynthetic pathway dependent on vitamin B6 (Figure 1).

Once produced or acquired from the diet, Trp can then be metabolized along different metabolic pathways (Figure 1). Humans mostly rely on Trp acquired from the diet as there is no evidence that Trp produced by bacteria could work as a Trp source [4]. Upon absorption by the gut, Trp is then transported to the liver via the hepatic portal system where the majority (around 90%) is metabolized in the kynurenine (Kyn) pathway that leads to the production of nicotinamide adenine dinucleotide (NAD^+^). Trp can also be metabolized in other cell types, such as endothelial cells, fibroblasts and immune cells [9]. The rate-limiting step in the Kyn pathway is represented by the enzymes indoleamine-2,3-dioxygenase 1 (IDO1), IDO2, and tryptophan-2,3-dioxygenase (TDO), which catabolize Trp to N’-formylkynurenine, which is then hydrolyzed to Kyn. Interestingly, although the dioxygenases IDOs and TDO do not use PLP as a cofactor, they can be considered indirectly dependent on PLP as they contain a heme group whose rate of synthesis is determined by the activity of the PLP-dependent enzyme 5-aminolevulinate synthase [10]. In particular, IDO1 is present in cells in the apo-form and can be activated upon addition of exogenous heme [11]. The role of heme binding for regulating IDO1 activity is also demonstrated by the finding that synthetic compounds targeting apoIDO1 are effective as inhibitors at both the protein and cellular levels [12]. In the path to the production of nicotinamide, Kyn is then converted to 3-hydroxykynurenine (3-hydroxykyn) by the activity of kynurenine hydroxylase (Kyn monooxygenase). Kyn and 3-hydroxykyn represent crucial branching points for the generation of Trp metabolites that depend on vitamin B6. Indeed, they can be substrates for the activity of two PLP-dependent enzymes, kynurenine aminotransferase (KAT) and kynureninase (KYNU). KAT converts Kyn and 3-hydroxykyn to kynureninate and xanthurenate, respectively, while anthranilate and 3-hydroxyanthranilate are generated by the activity of KYNU. 3-Hydroxyanthranilate can then be converted along the path to nicotinamide production while the other metabolites have distinct fates. The dependency of KYNU and KAT from PLP have led to the use of Trp metabolites of the Kyn pathway as potential markers of functional vitamin B6 status [13]. In particular, since KYNU is more susceptible to vitamin B6 deficiency than KAT, Trp metabolism is shifted towards increased production of xanthurenate [14]. In parallel, the levels of Kyn and 3-hydroxykyn are higher [15], likely dependent on increased flux of Trp along the Kyn pathway. By taking into account different parameters, including enzyme characteristics and metabolite abundances, the changes that occur upon B6 deficiency can be predicted by mathematical modelling [16]. Thus, analysis of Trp metabolites can provide accurate prediction of functional B6 status in health and disease [17,18].

Limited amounts of Trp may be used in other pathways such as for the production of the neuroactive molecule serotonin [2] (Figure 1). Interestingly, PLP-dependent activities also regulate the metabolic pathway that converts Trp to serotonin. Indeed, a Trp hydroxylase (Tph1, Tph2) first converts Trp to 5-hydroxytryptophan, which in turn is metabolized to serotonin by the PLP-dependent enzyme aromatic-L-amino acid decarboxylase (AADC). The metabolic dependence of serotonin from vitamin B6 has been evaluated in studies on the symptoms of depression. Indeed, it was first found that the levels of vitamin B6 were associated with depression [19], and it was then shown that vitamin B6 supplementation can protect from depression in certain clinical settings. All in all, these results indicate that the metabolism of Trp in mammals is dependent on vitamin B6 as two of the main Trp-metabolic pathways are regulated by PLP-dependent enzymes, thus making vitamin B6 a plausible metabolic checkpoint in the regulation of Trp flux.

A similar situation is also present in the microbial Trp metabolism (Figure 1). Dissection of the Trp metabolism in the gut microbiome has demonstrated the production of a wide variety of Trp metabolites [20]. The main pathways of Trp degradation include as a first step the production of indole, indole-3-pyruvic acid, tryptamine, and indole-acetamide [20]. The first three products are generated by the activity of PLP-dependent enzymes: indole is produced by tryptophanase, a PLP-enzyme that is activated by potassium ions; indole-3-pyruvic acid by PLP-dependent aromatic amino acid aminotransferases (AroAT); and tryptamine by a Trp decarboxylase, again dependent on PLP.

Collectively, these data indicate that vitamin B6 in the active form of PLP may represent a common denominator among the major pathways of Trp metabolism in both the host and microbes, thus suggesting that its levels may regulate the flux of Trp utilization and the generation of selective Trp metabolites.

## 3. A Biochemical Overview of Microbial and Host Trp-Metabolizing Enzymes

The main metabolic role of PLP-dependent enzymes is the synthesis, interconversion, and catabolism of amino acids, as well as the synthesis of secondary metabolites derived from amino acids, such as in particular neurotransmitters and hormones [21]. Notwithstanding their high versatility, PLP enzymes share some common features at both a functional and structural level [22,23]. The carbonyl group of the coenzyme forms a Schiff base linkage with the ε-amino group of an active site lysine residue, generating a complex called internal aldimine. Upon substrate binding, the internal aldimine is converted to the external aldimine, in which PLP is bound to the α-amino group of the amino acid substrate. The external aldimine intermediate is common to all PLP-catalyzed reactions, and it represents a crucial step to determine reaction specificity [24]. From the external aldimine, the reaction proceeds differently on the basis of which bond is broken at the Cα of the substrate, giving rise to transamination, racemization, decarboxylation, β-replacement, or β-elimination reactions [23,25] (Figure 2B). In each case, the positive charge of the pyridine nitrogen acts as an electron sink and stabilizes negatively charged reaction intermediates. According to the Dunathan hypothesis, the main determinants in controlling which one of the three labile bonds at Cα should be broken are stereoelectronic effects. They arise from the specific conformation of the external aldimine, so that the bond to be broken is that positioned perpendicular to the pyridine ring of the coenzyme. This happens because the resulting carbanion is better stabilized by resonance with the conjugated Π system due to the electronic delocalization of the negative charge [26]. The reaction and substrate specificity is dictated by the apoprotein, which drives the catalytic power of PLP toward the stabilization of different intermediates and favors the interaction with different ligands [24].

Due to their involvement in both microbial and host metabolism, PLP enzymes have become targets of clinical interest for different disorders including infectious diseases, neurologic diseases, cancer, and rare metabolic disorders [5,27,28,29,30]. This is especially true for members of the family involved in the different pathways of Trp metabolism, whose characteristics are described in the following sections.

### 3.1. Tryptophan Synthase (TS)

**Figure 3:**
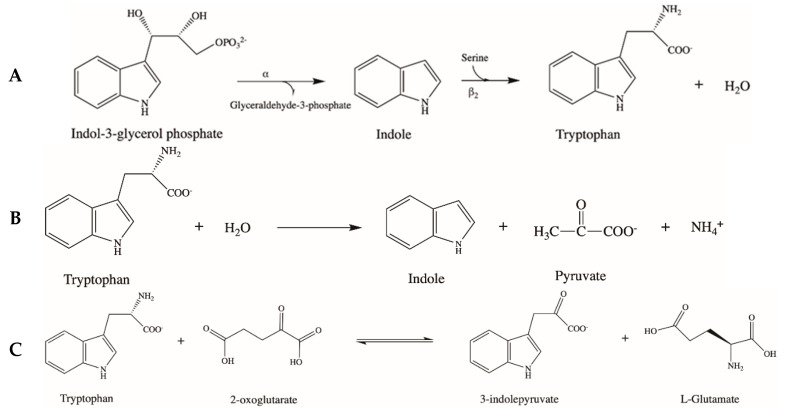

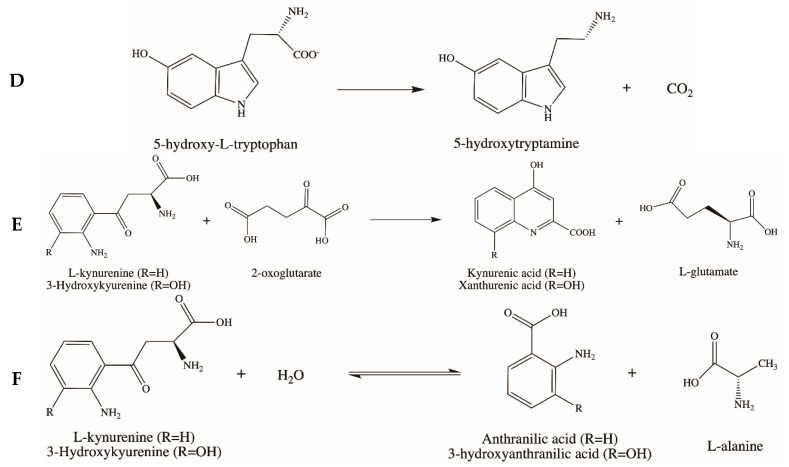
Schematic overview of the tryptophan metabolic pathways catalyzed by pyridoxal 5′-phosphate enzymes. The panel illustrates the substrates and the products of the reactions catalyzed by tryptophan synthase (TS) (**A**), tryptophan indole lyase (Trpase) (**B**), aromatic amino acid aminotransferases (AroAT) (**C**), aromatic-L-amino acid decarboxylase (AADC) (**D**), kynurenine aminotransferase (KAT) (**E**), and kynureninase (KYNU) (**F**). Details are described in the text.

TS (E.C. 4.1.2.20) catalyzes the last two steps of Trp synthesis in bacteria, yeast, molds, and plants [8] (Figure 3A). TS from *E. coli* and *S. typhimurium* are the most characterized enzymes and are often used as models for other homologous proteins [8,31,32]. The bacterial enzyme is an α_2_β_2_ tetrameric complex. Subunits α and β are encoded by the two adjacent genes of the *trp* operon, *trpA* and *trpB* [33], and catalyze two different reactions in a multi-step process. Subunit α converts indole-3-glycerol phosphate into glyceraldehyde 3-phosphate and indole by a reversible retro-aldol cleavage occurring through acid–base catalysis. The latter product, along with L-serine, is the substrate of subunit β, which utilizes PLP as a cofactor and catalyzes the β-replacement of the hydroxyl group of L-serine with the indole moiety, generating Trp [34]. At the β active site, the external aldimine with L-serine is first formed. Then, abstraction of the substrate α proton occurs, generating a quinonoid intermediate from which the hydroxyl group is eliminated giving the aminoacrylate species. The latter then reacts with indole leading to the Trp external aldimine passing through a second quinonoid intermediate [35]. α and β subunits are arranged in a αββα linear complex, with the α subunit displaying a TIM barrel conformation and the β subunit showing the typical arrangement of fold type II PLP enzymes [36]. An intriguing property of TS is substrate channeling. Indole, representing the product of the first reaction step and the substrate of the second one, is transferred from the α to the β subunit via an interconnecting tunnel. This explains why the two activities are structurally and functionally interconnected, and the formation of the αββα complex influences both catalytic efficiency and subunit switch from an open inactive to a closed active conformation [34]. The activity of TS β subunit is also allosterically regulated by monovalent cations, which increase catalytic efficiency by at least one order of magnitude, as well as by α subunit ligands [37,38].

The fact that Trp availability is essential for microbial survival and that the enzymes involved are absent in humans makes TS an attractive drug target for antibiotics development, although it should be underlined that Trp is essential only when dietary supply is low, thus explaining why some bacterial species maintain the *trp* operon at a very low level of functionality [33]. The regulation of the *trp* operon is under the control of two main signals, namely, the intracellular Trp concentration and the presence of charged/uncharged tRNA^Trp^ [33]. In Gram-negative bacteria, transcription is regulated by various mechanisms, including repression through a Trp-activated TrpR repressor, attenuation by sensing the presence of uncharged tRNA^Trp^, and response to the accumulation of Trp biosynthesis intermediates such as indole-glycerolphosphate. Similar signals are also sensed in Gram-positive bacteria but with different strategies. In *B. subtilis*, a Trp-activated trp RNA-binding attenuation protein (TRAP) is produced, whose activity is dependent on both Trp and tRNA^Trp^ levels, the latter through the production of an anti-TRAP protein [39]. In other Gram-positive bacteria, no TRAP protein is produced, but uncharged tRNA^Trp^ are sensed through a T-box mechanism [40]. TS expression is required for the survival of *M. tuberculosis* to counteract the adaptive immune response, and of other Gram-positive and Gram-negative bacteria [38]. In this regard, some active site ligands behaving as allosteric inhibitors of the *M. tuberculosis* enzyme have been identified through high-throughput screening and crystallographic studies [41,42]. The key role of Trp synthesis in *M. tuberculosis* is also demonstrated by the ability of microbiota-derived indole-propionic acid to inhibit bacterial growth in both in vitro and in vivo models [43,44]. As a Trp analog, indole-propionic acid binds an allosteric site of anthranilate synthase (TrpE), thus switching off Trp de novo synthesis by a regulatory feedback mechanism [45]. This action seems to be selective against mycobacteria, but the molecular reasons underlying this selectivity are currently unknown.

Notably, the resolution of the structure of TS β from *E. coli* in the holo- and apo-form has confirmed that the coenzyme also plays a structural role. Indeed, PLP binds very tightly (K_d_ ≈10 ^−7^ M), and strengthens the interaction between the two β subunits in the dimer, increasing their resistance to thermal stress. Moreover, it induces the transition from the open to the active closed form [46]. Thus, although Trp synthesis is known to be regulated by Trp itself by feedback inhibition mechanisms [33], the possibility exists that it could be also influenced by vitamin B6 availability.

### 3.2. Tryptophan Indole Lyase (Trpase)

The bacterial enzyme responsible for indole production is Trpase (E.C. 4.1.99.1). Trpase is a PLP enzyme that catalyzes a reversible β-elimination reaction converting L-Trp to indole, pyruvate, and ammonium ion (Figure 3B). In *E. coli*, the enzyme is encoded by the *tnaA* gene of the *tna* operon, which also comprises a Trp-specific membrane transporter for Trp uptake from the extracellular medium encoded by the *tnaB* gene [47]. The detailed kinetic characterization of the Trpase reaction has indicated that it consists of an electrophilic aromatic substitution occurring through a S_E_2 mechanism. Upon Trp binding and external aldimine formation, the Cα-proton of the substrate is abstracted, generating a quinonoid intermediate. The concerted reprotonation at Cβ/deprotonation of the aromatic ring leads to the formation of indole and of an aminoacrylate intermediate. The subsequent transaldimination regenerates the PLP form of the enzyme and iminopyruvate, which is hydrolyzed to pyruvate and ammonium ion outside of the active site [48]. In vitro Trpase shows a wide substrate specificity, being able to catalyze elimination reactions with different amino acid substrates. In addition, amino acid compounds that cannot undergo elimination behave as competitive inhibitors of the enzyme [49]. The crystal structure of Trpase from *Proteus vulgaris* solved in 1998 [50] has revealed that it displays a tetrameric assembly and belongs to the Fold Type I family, the most numerous group of PLP enzymes. Each subunit is formed by a N-terminal stretch, a large domain comprising most of the active site, and a small C-terminal domain. As in all members of the same family, each active site is located in a pocket between the large and small domain made up of residues belonging to two different subunits of a dimeric unit [51]. Monovalent cations influence Trpase tetramer stability and PLP binding, by possibly forcing each subunit in a proper conformation for catalysis [52].

Data obtained in *E. coli* have clearly demonstrated that the concerted action of Trpase and the TnaB transporter produce millimolar amounts of indole from exogenous Trp. This production mainly occurs when high amounts of Trp are available, such as in the intestinal tract, where specific niches for *E. coli* can form and give rise to high local intestinal indole concentrations [53]. More recently, it has been reported that the *trp* operon displays a bistable behavior, and that the activity of Trpase can be influenced by both glucose and Trp extracellular levels through a common signaling pathway [47]. No studies have been performed until now to define if the activity is also dependent on the levels of coenzyme. It is generally assumed that the levels of vitamin B6 can influence the intracellular levels of active PLP enzymes in different ways, including the change in the proportion of apoenzymes, which are often more prone to degradation than the corresponding holoenzymes [54]. Considering that Trpase is endowed with a relatively low affinity for PLP, the intracellular PLP concentration is in the micromolar range [55], and apoTrpase undergoes cold inactivation [48], the possibility that Trpase levels and activity could be influenced by vitamin B6 merits investigation.

### 3.3. Aromatic Amino Acid Aminotransferase (AroAT)

Enzymes named AroAT (E.C. 2.6.1.57) catalyze a reversible reaction converting Phe, Tyr, and/or Trp to the corresponding α-keto acids (Figure 3C). The overall transamination occurs through a ping-pong mechanism and can be described as two half-reactions. In the first half-reaction, the amino donor binds to the enzyme in the PLP form, generating the external aldimine. Then, a proton shift from Cα of the substrate moiety to the C4′ of the cofactor occurs, producing a ketimine intermediate, generating the α-keto acid product and the enzyme with bound pyridoxamine phosphate (PMP). In the second half-reaction, the amino acceptor substrate binds to the PMP-bound enzyme, generating a second ketimine from which an inverse proton transfer from C4′ to Cα occurs, producing the external aldimine of PLP with the α-amino acid product that is finally released [30,56].

AroATs are expressed by different organisms, including bacteria, plants, and fungi (BRENDA database, www.brenda-enzymes.org/). An important feature of these enzymes is their broad substrate specificity, being able to use also dicarboxylic amino and oxo-acids as substrates [57], which is paired by their functional heterogeneity in terms of metabolic role. AroAT from plants are highly specific for Trp transamination, as a step for the synthesis of the plant hormone indoleacetic acid [58]. In bacteria, they are mostly involved in the synthesis of Phe and Tyr from the corresponding oxoacids [59,60]. AroAT from *E. coli* displays a K_m_ for Trp one order of magnitude higher than that for Tyr and Phe [61]. In *Pseudomonas*, different genes encoding AroATs are present with overlapping metabolic activity, as shown by the fact that the mutation of one of them does not lead to auxotrophy for Phe or Tyr [62]. Interestingly, an AroAT from *Kleibsiella aerogenes* is involved in the use of Trp as a carbon source. No homologous enzyme is expressed by *E. coli* [59]. In the common gut bacterium *Clostridium sporogenes*, Trp transamination by AroAT is the first step for the production of various metabolically active compounds including indole pyruvic acid, which accumulates in the host serum and probably mediates the microbe–host interactions regulating immune, metabolic, and neuronal responses [43,63]. In fungi, the *Aro8* gene encodes a versatile PLP-dependent AroAT whose metabolic role is shared between the lysine synthesis by the aminoadipate pathway and the catabolism of aromatic amino acids [64,65]. In *C. albicans*, Aro8 is a very versatile enzyme active against L-histidine, L-lysine, and aromatic amino acids [64]. Aro8 from *S. cerevisiae* has been characterized both functionally and structurally and has been found to be a homodimer belonging to fold type I that is able to use aromatic amino acids as well as α-aminoadipate as substrates [66,67]. Interestingly, it shows a broad substrate specificity and its role in KA production has been demonstrated [68]. An enzyme able to transaminate Trp has also been identified in crude rat liver extracts, although it has been found that most of the activity can be ascribed to the action of other enzymes, mainly tyrosine aminotransferase [69].

In general, AroAT have greater affinity for Phe and Tyr when compared to Trp; thus, their role in Trp metabolism is probably largely dependent on Trp availability. Moreover, they bind PLP in a very tight manner, as usually observed for aminotransferases [23], thus suggesting that their activity would not be modulated by vitamin B6 levels. Nevertheless, since Trp transamination represents a key step for the production of microbial metabolites that regulate host physiology, deciphering how it is modulated by vitamin B6 levels could reveal unknown mechanisms related to the pathogenesis of human diseases and possibly open therapeutic avenues.

### 3.4. Aromatic Amino Acid Decarboxylase (AADC)

The presence of PLP-dependent enzymes able to catalyze the decarboxylation of either Trp or Trp-derived metabolites, namely AADC, has been described in both host and microbes. PLP-dependent decarboxylation occurs when, upon substrate binding, the carboxyl group is released as CO_2_ producing a quinonoid intermediate. The latter is then reprotonated at Cα, generating the amine product [70] (Figure 3D). Most of the biochemical features known on AADC until now are related to the enzyme from pig kidney, which shares 90% of sequence identity with the human counterpart. Spectroscopic, kinetic, and mutagenesis studies have dissected the catalytic mechanism, discovered how reaction specificity is dictated by the presence or absence of oxygen, and identified specific active site inhibitors [71,72,73,74,75]. Structurally, both microbial and mammalian AADC are homodimers and belong to the fold type I family. Each subunit is formed by a large domain made up of a β/α -barrel that contains the PLP binding site and a C-terminal small domain. The monomer–monomer interface is very large because of the presence of an N-terminal domain generating two α-helices that pack over the opposite subunit [73,76]. AADC displays high affinity for PLP [77], which also plays an important structural role for the protein. In fact, the apo-form of human AADC shows an open conformation resulting from a 20 Å movement of each subunit leading to the exposure of the active site to the solvent [78]. One of the most important consequences of this structural change is the increased sensitivity of the apoenzyme to degradation, as observed in rat brain cells [79]. This has led to the hypothesis that PLP availability could influence the amount of functional enzyme present in the brain [78].

Although microbial and host AADC seem to share most of their biochemical features, their substrate preferences and physiological role seem different. Human AADC is the enzyme responsible for dopamine and serotonin biosynthesis, starting from the corresponding amino acids L-dopa and 5-hydroxyTrp, respectively [70]. AADC is expressed in both neuronal and non-neuronal tissues, with the latter being found mainly in the kidneys and in the gastrointestinal tract [80]. In the gut, enteroendocrine cells called enterochromaffin cells play a sensor role for stimuli coming from the lumen and are mainly responsible for serotonin production. Serotonin produced in the gut mediates a variety of processes including peristalsis, secretion, vasodilation, and perception of pain or nausea [80]. An altered production of serotonin in the gut is also observed in several pathologic conditions related to inflammation, such as inflammatory bowel disease, colitis, and celiac disease. It has been demonstrated in various experimental settings that the rate-limiting step of serotonin synthesis is Trp hydroxylation in cerebral tissues. The latter activity is mainly influenced by Trp availability, because of the low K_m_ of the hydroxylase for the substrate [81]. However, it has been shown that neuronal AADC activity is reduced in the case of vitamin B6 deficiency, either as a consequence of inherited conditions or as a side-effect of pharmacological treatments [82]. Thus, the decarboxylation step can become limiting under conditions of PLP deficiency in the CNS [83,84]. Whether a similar regulation also occurs at intestinal level remains to be defined.

The presence of a microbial enzyme showing AADC activity has been also found in 2014 by Williams and co-authors [76], who described a PLP enzyme produced by the two human commensals *Clostridium sporogenes* and *Ruminococcus gnavus*, showing Trp decarboxylating activity that generates tryptamine. These enzymes show a preference for Trp over Phe and Tyr, and excrete tryptamine in the extracellular medium. Interestingly, in silico data suggest that homologues of these enzymes could be present in about 10% of the human microbiome [76]. The production of tryptamine by the gut microbiome represents a very important route of Trp metabolism that could influence host metabolism. Indeed, tryptamine levels upon colonization of germ-free mice with human stool increases by about 200-fold. Recently, it has been demonstrated that bacterially produced tryptamine accelerates gastrointestinal transit through stimulation of GPCR receptors located in the intestinal epithelium [85]. In cerebrospinal fluid, tryptamine produced by AADC is the precursor of IAA, one of the Trp catabolites involved in the regulation of the gut–brain axis [86,87]. Thus, host physiology is influenced by a bacterial product of Trp metabolism, therein further supporting the pivotal role of PLP enzymes as therapeutic targets at the host–microbe interface.

### 3.5. Kynurenine Aminotransferase (KAT) and Kynureninase (KYNU)

The Kyn pathway (KP) is one of the major players in determining the plasmatic levels of circulating Trp and of Trp metabolites, being responsible for the fate of about 95% of Trp. The KP is present in both the liver and peripheral tissues, serving different roles including the synthesis of NAD^+^ and other intermediate bioactive compounds with important immunomodulatory functions [9]. Two PLP-dependent enzymes are involved in the KP, namely, KAT and KYNU.

KAT (E.C. 2.6.1.7) catalyzes the irreversible conversion of Kyn to kynurenic acid (KA) (Figure 3E), an important neuroactive compound known to behave as an endogenous antagonist that binds the glycine site in NMDA receptors [88], as well as agonist of receptors involved in immunomodulation such as aryl hydrocarbon receptor (AhR) [89]. The enzyme also converts 3-hydroxykyn to xanthurenic acid (XA) (Figure 3E), thus controlling the metabolic flux of the KP. The reaction consists of a classical PLP-dependent transamination occurring through a ping-pong mechanism converting the amino acid substrate, Kyn, into the corresponding α-ketoacid that spontaneously cyclizes to form KA and the enzyme in the PMP form. The latter can then transfer the aminic group to an α-ketoacid, forming the corresponding amino acid and regenerating PLP-KAT to complete the reaction cycle [23]. Four different genes encoding KAT have been characterized in humans and rodents, which are named KAT I, II, III, and IV [90]. The four isoenzymes are phylogenetically linked, and share many biochemical features, but also show specific properties in terms of expression and catalytic efficiency toward Kyn and 3-hydroxykyn [90]. KAT I and KAT III share the highest sequence identity. Both are multi-functional enzymes active toward a variety of α-amino acids (preferentially those with large neutral, aromatic, or sulfur-containing side chains) and α-ketoacids [91,92]. KAT II is known for its unique ability to use α-aminoadipate as an amino donor, and it also uses α-ketoglutarate as an amino acceptor [93]. It is not active against 3-hydroxykyn. In bacteria and fungi, the enzyme is also involved in the lysine metabolism [94,95]. KAT II is the most abundant isoenzyme in the human brain and is considered to be mainly responsible for KA synthesis [96]. KAT III is also known as glutamine transaminase and has only recently been included among KAT isoenzymes [97]. It shows cysteine conjugate β-lyase activity and is abundantly expressed in the kidney and liver [98]. KAT IV is the most conserved isoenzyme, active toward a plethora of amino and keto acids [99]. It displays aspartate aminotransferase activity, and therefore is important for the transfer of NADH, reducing power from cytosol to mitochondria. Moreover, it plays a key role for glutamate synthesis in the brain and is important for astrocytes metabolism [100].

The crystal structures of the four KAT isoenzymes are available from different sources, including that of human KAT I and KAT II, as well as of mouse KAT III and KAT IV [101]. All KATs belong to the fold type I family, and are functionally obligated homodimers, being the active site comprised between the two subunits. Although the solved structures are very similar, some key peculiar features of each isoenzyme can be evidenced that mainly relate to the N-terminus of each monomer, which shapes the ligand binding pocket. In KAT I, the N-terminus closes the entrance of the active site upon ligand binding, mainly through the precise movement of Trp18, generating a relatively rigid pocket that accommodates the aromatic substrate [102]. KAT III shows very similar features and mainly differs for the residues located at the entrance of the active site, which in turn can determine the different substrate specificities of the two proteins [92]. The structure of KAT IV is very similar to that of mitochondrial aspartate aminotransferase and reveals a conformational change occurring upon substrate binding involving the small domain that has the role of shielding the active site from the solvent [99]. From a structural point of view, KAT II merits a special mention, because of a domain swapping of the N-terminus of one subunit to the opposite one, which is different depending on the chemical features of the ligand and explains the wide substrate specificity of this isoenzyme [103]. It is worth noting that the differential structural features of the KATs have prompted the identification of inhibitors specific for a particular isoenzyme as putative therapeutic approaches for CNS diseases [104].

KYNU (E.C. 3.7.1.3) catalyzes the hydrolytic cleavage of L-Kyn or 3-hydroxykyn to anthranilic acid or 3-hydroxyanthranilic acid, respectively, as well as L-alanine [105] (Figure 3F). The enzyme is expressed in both prokaryotes and eukaryotes. In bacteria, Kyn is the preferred substrate, and the reaction probably plays an anabolic role for species that utilize Trp as a metabolic fuel. On the other hand, eukaryotic KYNU is mainly active on 3-hydroxykyn and the Trp degradation pathway in the liver allows the synthesis of NAD^+^ under conditions in which there is the need of niacin or the production of quinolinic acid in immune cells [106,107]. The kinetic and structural features of KYNU from different sources, including humans and *Pseudomonas fluorescens*, have been widely investigated [106,107]. KYNU is a dimeric fold type I protein and shares many structural features with aminotransferases, including key active site residues. The comparison between prokaryotic and eukaryotic enzymes has also allowed for the identification of residues that govern substrate preferences [108,109]. KYNU reaction occurs through a mechanism based on acid/base catalysis. The bound substrate is first deprotonated at Cα and then reprotonated at C4′, generating a ketimine intermediate. The addition of water to the ketimine, a step in which the phosphate of PLP plays an important role, generates a *gem*-diol. The latter intermediate then undergoes C_β_–C_γ_ bond cleavage to give the carboxylic acid product and an enamine intermediate. The subsequent protonation of the enamine produces a pyruvate ketimine, which after deprotonation at C4′ and reprotonation at Cα finally regenerates the enzyme in the PLP form and the second product alanine [106,107].

The pivotal role of PLP enzymes in the kynurenine pathway explains why it is sensitive to alterations of the vitamin B6 status, as previously described.

## 4. Vitamin B6 and the Regulation of Trp Flux in the Host and Microbes

**Figure 4:**
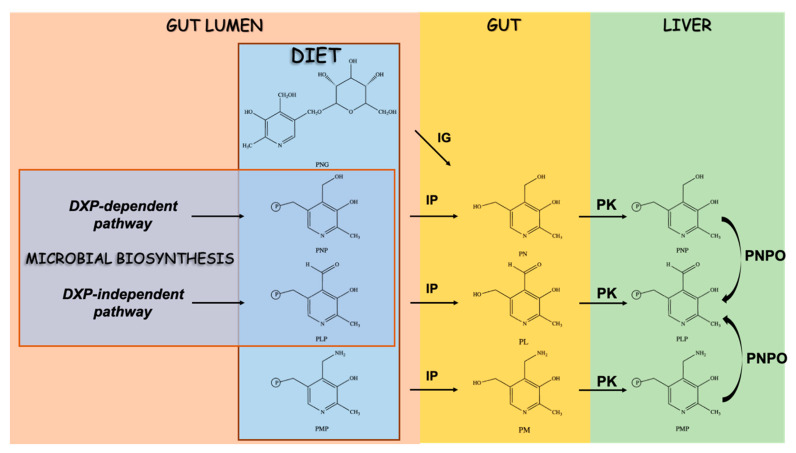
Schematic overview of the microbial and host B6 vitamers metabolic pathways. The panel depicts the pathways of B6 vitamer metabolism in the gut microbiota (pink, left side) and in the host, with particular reference to the gut (yellow, central) and the liver (green, right side) metabolic pathways. The panel highlights the different contribution of the microbiome and the diet to the host vitamin B6. Details are described in the text. DXP: deoxyxylulose 5-phosphate; IG: intestinal glycosidase; IP: intestinal phosphatase; PK: pyridoxal kinase; PL: pyridoxal; PLP: pyridoxal 5′-phosphate; PM: pyridoxamine; PMP: pyridoxamine 5′-phosphate; PN: pyridoxine; PNG: pyridoxine glucoside; PNP: pyridoxine phosphate; PNPO: pyridox(am)ine phosphate oxidase.

In humans, vitamin B6 is acquired from the diet in the form of pyridoxine (PN) and its phosphate (PNP) and glucoside (PNG) derivatives, PLP and pyridoxamine 5′-phosphate (PMP) [5] (Figure 1 and Figure 4).

The phosphorylated forms PNP, PLP, and PMP are dephosphorylated by intestinal phosphatases while PNG is hydrolyzed by an intestinal glycosidase before absorption by intestinal cells. The resulting PN, pyridoxal (PL), and pyridoxamine (PM) vitamers are then converted in the intestine or in the liver (primary site) to the phosphorylated forms by the activity of pyridoxal kinase that generates PNP, PLP, and PMP. Finally, pyridox(am)ine phosphate oxidase (PNPO) converts PNP and PMP to PLP, which is then exported to the other tissues upon binding to lysine 190 of albumin [5]. When tissue distribution was evaluated in mice fed different concentrations of PN, a great difference was found among several tissues in their levels of B6 vitamers [110]. Interestingly, a dose-dependent accumulation of PN and PLP was observed only in the small intestine and colon, but not in other organs [110], indicating that intestinal cells can absorb and partially produce PLP in direct response to dietary intake (Figure 1 and Figure 4). In addition to the diet, vitamin B6 can also be produced by the microbiome in the form of PLP [111] (Figure 1 and Figure 4)**.** In particular, two biosynthetic pathways have been described, the DPX-dependent and DPX-independent pathways leading to PNP and PLP, respectively [112], which associate with different phyla in the gut microbiome [111]. The microbial contribution to human B6 requirements is consistent, such that dietary B6 deficiencies are rare [5]. Interestingly, the dietary consumption of vitamins can influence the composition and structure of the colon microbiome [113]. In particular, a higher dietary consumption of vitamin B6, among others, was associated with a greater richness and evenness of the gut microbiota [113]. Therefore, the gut represents the site for the integration of dietary and microbial vitamin B6 sources that results in the adjustment of the B6 content of intestinal cells and the composition of its associated microbiome.

As a corollary, the Trp metabolism by PLP-dependent enzymes in the gut may reflect the vitamin B6 levels, and the production of Trp metabolites may vary accordingly. Indeed, a variation in the availability of PLP is expected to modulate the production of Trp by the TS. We have previously shown that the levels of Trp condition the host–fungal crosstalk via the microbiome [114]. In conditions of unrestricted Trp availability, such as in IDO1-deficiency, *Lactobacilli* expand and produce an indole derivative, indole-3-aldehyde (3-IAld), which activates the aryl hydrocarbon receptor (AhR) to produce IL-22 and protect the intestinal barrier. On the contrary, in IDO1 sufficiency, the IDO1 pathway in epithelial and dendritic cells prevail with the production of Kyn, the generation of Treg, and the production of IL-10 [114]. Altered vitamin B6 levels may mimic this scenario. Indeed, the levels of PLP not only influence the synthesis of Trp, but also the activity of AroAT and the generation of its substrate indole-3-pyruvate, a potential precursor of 3-IAld, while the activity of IDO1 is not dependent on PLP and the synthesis of Kyn may occur unperturbed. Therefore, vitamin B6 appears as a metabolic checkpoint that orientates the flux of Trp in the host and the microbes to influence the immune response to external challenges. The same role of vitamin B6 in the regulation of host–fungal interaction mediated by the microbiota may apply to other tissues as well. Indeed, we have recently performed a multicenter, prospective, observational study termed SNIF (Survey of Nasal InFection) in which hematological patients were enrolled and their nasal and pharyngeal microbiomes were characterized, on average, monthly from the enrollment and up to 6 months (manuscript submitted). Upon stratification of samples according to their risk of developing invasive fungal infections on the basis of an algorithm developed by the SEIFEM (Epidemiology Survey of Invasive Fungal Infections in Hematological Malignancies) [115], the composition and the metabolic profile of the pharyngeal microbiome was compared between low-risk and high-risk groups. Among others, the PLP biosynthesis and salvage pathway was associated with the low-risk group and, in parallel, the levels of Trp and 3-IAld were significantly higher in the low-risk than the high-risk group while Kyn levels were similar. Thus, in analogy with the results obtained in the gut, the crosstalk between the host and fungi might be regulated by PLP levels dictated by the microbiome that, in turn, influence the flux of Trp in the host and microbes. Of note, 3-IAld administration protected from infection and inflammation in a mouse model of aspergillosis (manuscript submitted), supporting the notion that correcting the Trp flux might provide beneficial effects.

Other examples of microbiome influence on Trp metabolic pathways dependent on vitamin B6 may be hypothesized. For instance, vitamin B6 levels may modulate the production of serotonin by enterochromaffin cells. It has been reported that the gut microbiome stimulates the synthesis of serotonin by producing metabolites that directly stimulate colonic ECs to produce serotonin [116]. In addition to the proposed mechanism, the requirement for the microbiome might also be linked to the production of vitamin B6 that is required for the PLP-dependent activity of AADC that converts 5-hydroxytryptophan to serotonin. It is interesting to note that, while vitamin B6 may modulate Trp metabolism by influencing the activity of PLP-dependent enzymes, Trp metabolites can influence vitamin B6 metabolism as well [117].

In conclusion, vitamin B6 and Trp synthesis and metabolism in the gut and other tissues may crosstalk by intersecting multiple host and microbial pathways with potential implications in the regulation of the mucosal immune response and the synthesis of bioactive molecules.

## 5. Vitamin B6 Dysregulation in Diseases of the Gastrointestinal Tract

Since vitamin B6 may regulate crucial physiological events associated with Trp metabolism in the host and the microbes, the possibility exists that a dysregulation in the levels of vitamin B6 results in pathological conditions. An elegant perspective on the role of Trp metabolism in the crosstalk between the host and the microbiome in health and disease has been recently reviewed [4]. The authors discuss how a disequilibrium between the serotonin, Kyn-IDO, and indole-AhR pathways might be causative of pathological conditions and that re-establishing the correct Trp flux could represent a therapeutic approach. For instance, on the one hand, Crohn’s disease (CD) and ulcerative colitis (UC) are characterized by a reduced AhR pathway, and AhR agonists or AhR-activating probiotics might be beneficial. On the other hand, irritable bowel syndrome (IBS) with constipation and IBS with diarrhea are characterized by a reduced or increased Tph1 pathway, respectively, and, accordingly, 5-HT4 receptor agonists or 5-HT3 receptor antagonists are suggested as therapy [4]. We can hypothesize that re-establishing vitamin B6 homeostasis might re-equilibrate the flux along the different metabolic pathways by restoring the proper functional activities of the PLP-dependent enzymes that populate each pathway. For instance, patients with active CD and UC have significantly lower plasma PLP concentrations than patients with quiescent disease or healthy controls [118] and an inverse relationship has been shown between vitamin B6 intake and the severity of IBS symptoms [119]. Similarly, patients with celiac disease are reported as vitamin B6-deficient [120,121]. This would suggest that dietary supplementation with vitamin B6 might be therapeutic. However, this clinical transition might not be straightforward. For instance, study in animal models of inflammatory bowel disease have provided unpredictable results [122]. Indeed, B6 deficiency was initially shown to be protective in DSS-induced colitis [123] while a subsequent study reported a bell-shaped response curve in which moderate vitamin B6 supplementation and mild depletion significantly reduced colonic inflammation [124]. This variability might be related to the wide variety of enzymatic reactions to which PLP participates, as well as to the different repartitioning of the Trp flux in different organs and tissues. For instance, the Trp flux, as determined by Trp availability, quality, and quantity of PLP-dependent enzymes, and composition and metabolic profile of the microbiome, might be different such that modulating the vitamin B6 levels systemically might impact the Trp flux at distinct sites with opposite outcomes. A vitamin B6-based approach requires a local characterization of PLP-dependent reactions and a proper calibration, either by modulating the microbiome or selected host enzymatic activities, in order to redirect the flux of Trp where it is needed and optimize the efficacy/safety profile.

## 6. Vitamin B6 Dysregulation in Immunology

The immune system is highly dependent on vitamin availability for the control and prevention of infections as opportunistic infections or in general to control immune escape in terms of cancer cells. In particular, vitamin B6 deficiency is very common in the field of malnutrition and it is known that it may severely impact the fitness of the host immune system [125]. In general, vitamin B6 deficiency has been associated with different diseases, leading to autoinflammation as well as cancer development. First studies supported the idea that B6 deficiency would lead to immunocompromision as for the impact on T cell polarization or antibody production [126].

In addition, several studies highlight that vitamin B6 deficiency and consequently PLP low plasma levels inversely correlate with inflammatory markers as protein C reactive, but highly predispose to systemic inflammation as coronary artery disease, atherosclerosis, and rheumatoid arthritis [127,128].

More recently, mechanistically speaking, vitamin B6 has been demonstrated to reduce IL-1β release by macrophages. These results may also explain how vitamin B6 deficiency may chronically activate NALP3 inflammasome and predispose an individual to inflammatory diseases [129].

Interestingly, in patients with rheumatoid arthritis, a therapy supported by vitamin B6 supplementation (100 mg/day) reduces plasma IL-6 and TNF-α cytokine production.

However, the anti-inflammatory mechanisms of vitamin B6 are not totally clarified in the immunological fields. A novel hypothesis may relate vitamin B6 and Trp catabolism to inflammatory status [130], knowing that two major Trp catabolic enzymes, IDO or Tph1, are especially known to regulate peripheral tolerance in infections or autoimmune diseases [131].

In the GI tract, serotonin is synthesized by specialized endocrine cells, called enterochromaffin cells, as well as mucosal mast cells [132] and myenteric neurons [133]. *Tph1* was also found to be expressed in Th0, Th1, Th2, and iTreg-polarized cells [131]. Interestingly, Tph1 activation by NAD+ was found to protect against multiple sclerosis, an autoimmune disease where autoreactive T cells progressively react against self-antigens [131]. In this study, authors found that Tph1 was able to regulate T cell polarization and ameliorate autoimmune disease in mice. This may underline the importance of vitamin B6 in T cell polarization and peripheral control of inflammation [131].

To further confirm this consideration, clinical studies also proved that vitamin B6 may also regulate myelination and remyelination in multiple sclerosis patients [134].

Thus, few studies were focused on the role of vitamin B6 on peripheral tolerance, which might be very important since both Tph1 and IDO were demonstrated to be players of tolerogenic immune responses. Indeed, in opportunistic diseases, where tolerance is “protective” for immune functions [135], vitamin B6 may definitively regulate the whole process.

## 7. Conclusions

The complex network of host and microbial metabolic pathways centered around Trp outlines the importance of Trp metabolites in different physiological processes. The comprehension of the relationships that keep the whole network together opens up great opportunities for therapeutic interventions, but solving the puzzle is a challenging task. In the present opinion, we provided an interpretative key to this network by highlighting the critical role played by PLP-dependent enzymes in Trp metabolism. The description of each enzyme along with its dependence on PLP could represent a starting point for the elaboration of an algorithm that takes into account the local concentration of vitamin B6 and the specific host and microbial PLP enzymes to predict the direction of Trp flux and the relative proportion of Trp metabolites that will be generated. Although much work is needed to delineate the Trp metabolic network under this perspective, we believe that placing emphasis on vitamin B6 might direct future work on Trp metabolism in health and disease.

## Abbreviations

3-hydroxykyn	3-hydroxykynurenine
5-hydroxyTrp	5-hydroxytryptophan
AroAT	aromatic amino acid aminotransferases
AADC	aromatic-L-amino acid decarboxylase
AhR	aryl hydrocarbon receptor
CD	Crohn’s disease
CRISPR	clustered regularly interspaced short palindromic repeats
Cas9	CRISPR-associated protein-9 nuclease
RT-PCR	real-time reverse transcription-polymerase chain reaction
ELISA	enzyme-linked immunosorbent assay
NF-κB	nuclear factor kappa-light-chain-enhancer of activated B cells
PAK1	p21-activated kinase 1
FAK	focal adhesion kinase
SDS-PAGE	sodium dodecyl sulfate polyacrylamide gel electrophoresis
HRP	horseradish peroxidase
EC	enterochromaffin cells
3-IAld	indole-3-aldehyde
IDO	indoleamine-2,3-dioxygenase
IBS	irritable bowel syndrome
KYNU	kynureninase
KA	kynurenic acid
Kyn	kynurenine
KAT	kynurenine aminotransferase
KP	kynurenine pathway
PL	pyridoxal
PM	pyridoxamine
PMP	pyridoxamine phosphate
PN	pyridoxine
PNG	pyridoxine glucoside
PNP	pyridoxine phosphate
TDO	tryptophan-2,3-dioxygenase
Tph	tryptophan hydroxylase
Trpase	tryptophan indole lyase
TS	tryptophan synthase
UC	ulcerative colitis
XA	xanthurenic acid
